# Perceived teacher support and student engagement among higher education students – a systematic literature review

**DOI:** 10.1186/s40359-025-02412-w

**Published:** 2025-02-11

**Authors:** Kartika Prananto, Surya Cahyadi, Fitriani Yustikasari Lubis, Zahrotur Rusyda Hinduan

**Affiliations:** 1 Psychology Doctoral Study Program, Faculty of Psychology, Universitas Padjadjaran, Jatinangor, Indonesia; 2 Center for Psychological Innovation and Research, Faculty of Psychology, Universitas Padjadjaran, Jatinangor, Indonesia

**Keywords:** Teacher-student relationships, Supportive teaching practices, Student active participation, Mediating factors, Moderating factors, Theoretical framework, Methodology

## Abstract

**Background:**

Research on student engagement has garnered significant interest from educators and practitioners because of its direct impact on academic success and achievement. Engaged students tend to perform better academically and exhibit fewer undesirable study behaviors, thereby enhancing academic outcomes.

**Objective:**

This systematic literature review consolidates research on the impact of perceived teacher support on student engagement in higher education. This study emphasizes the association between teacher support in improving students’ academic performance, motivation, and retention. Furthermore, the review explores key theoretical frameworks, such as self-determination theory and social cognitive theory, alongside methodological tools such as measurement instruments and statistical analyses. The goal is to equip psychologists and educational researchers with insights into the relevant frameworks, tools, and methods for advancing future studies within the context of higher education.

**Methods:**

This study followed the Preferred Reporting Items for Systematic Reviews and Meta-Analyses (PRISMA) methodology. We conducted a comprehensive search for academic studies published in English within databases such as APA PsycNet, Scopus, ERIC, EBSCOHost, ProQuest, and PubMed to identify eligible studies published between 2014 and 2024.

**Results:**

A review of 13 selected articles revealed that both students’ personal characteristics and school environment factors mediate and moderate the relationship between perceived teacher support and student engagement. The students’ personal characteristics factors include self-efficacy, the fulfillment of psychological needs, and motivation, whereas school environment factors involve the learning environment and the quality of teacher-student and peer relationships. Our findings show a lack of studies prior to 2020, with most research conducted in China and limited contributions from Malaysia and Vietnam. The reviewed articles predominantly used cross-sectional quantitative designs and self-report questionnaires, employing statistical methods like path analysis and structural equation modeling. Theoretical frameworks on student engagement mostly followed Fredricks et al.‘s model, while teacher support theories varied, with three main patterns identified: direct influence, mediation through basic psychological needs, and social cognitive perspectives. This review emphasizes the crucial role of teacher support in enhancing student engagement in higher education and urges further exploration in this under-researched area.

**Conclusion:**

In conclusion, this review underscores the significant role of teacher support in enhancing student engagement in higher education. It highlights key theoretical frameworks and research methodologies, offering valuable insights for future studies aimed at advancing teacher support and student engagement in this context.

## Introduction

 The concept of student engagement has gained significant attention as a research focus in education over the past several decades. In this paper, student engagement is defined as “the time and effort students invest in activities that are empirically associated with favorable college outcomes and the actions institutions take to encourage student participation in these activities” [1]. Student engagement is a malleable concept that involves the interplay between the individual and their environment. Research on student engagement highlights a range of advantages that support academic success in higher education. These include improved academic achievement, a greater probability of degree completion, lower risks of burnout and dropout, and a reduction in negative behaviors within academic environments [2–5]. Given its significant positive impact on student learning outcomes, student engagement has been integrated into a variety of interventions and educational objectives [3].

Extensive research conceptualizes student engagement as an outcome stemming from motivational processes. The motivation cultivated within students functions as a pivotal force, fostering their active engagement in the learning process [6]. Appleton [3] broadens this perspective by highlighting that engagement is intrinsically relational [7]; it extends beyond individual effort [8], necessitating an alignment between the individual and their environment [9].. However, ​​Furrer et al. [10] highlighted the necessity of understanding engagement within a motivational framework, noting that engagement can evolve through cyclic interactions with contextual variables and subsequently impact academic, behavioral, and social outcomes influenced by these changes in engagement. This perspective aligns with Ryan and Deci’s framework [11], which differentiates between intrinsic and extrinsic motivation sources and examines how social influences can enhance or hinder the quality of individual performance.

Multiple studies have indicated that social support, particularly from teachers, plays a crucial role in fostering student engagement [12–18]. Research by Li and Xue [19] has demonstrated that both the quality of teacher-student relationships and positive teacher behaviors are moderately correlated with student engagement. Strong teacher-student relationships contribute to a relaxed and enjoyable learning environment for students. Furthermore, constructive teacher behaviors—such as offering guidance, fostering motivation, providing timely feedback, and demonstrating other forms of support—can substantially enhance student engagement. Quin [20] also identified a connection between teacher-student relationships and student engagement.

To our knowledge, systematic reviews on perceived teacher support and student engagement remain relatively limited. A review addressed the relationship between teacher support and student engagement, specifically within the context of physical education [21]. This research revealed a positive correlation between perceived teacher support and student engagement in physical education. Most of the study samples are from secondary and high school students, focusing primarily on a specific subject area, such as the foreign language. Therefore, further research involving higher education populations and general learning activities is necessary to validate these findings. Additionally, another review concentrated on teacher autonomy support [22]. This research shows, in line with self-determination theory (SDT) [11], that students’ motivation and engagement in the classroom are influenced by their perceptions of the learning environment and how teachers fulfill their basic psychological needs. However, this study focused exclusively on autonomy support. However, aspects of structure and involvement could be explored to obtain a more complete understanding of teacher support. Although the positive impact of teacher support on student engagement has been widely acknowledged in the scholarly literature, there is a notable gap in comprehensive reviews that address all aspects of teacher support within the context of higher education. According to the self-system model of motivational development (SSMMD), teacher support manifested through autonomy, structure and involvement promotes student engagement by fulfilling basic psychological needs [23]. However, some studies argue that this relationship is indirect yet significant [24]. This perspective contrasts with the findings of previous studies [18, 19, 25, 26] which suggest that college students’ perceptions of teacher support have a direct and significant positive effect on student engagement.

Accordingly, this study adopts a systematic approach to perform a comprehensive literature search and review of empirical evidence, aiming to identify factors that mediate and moderate the relationship between perceived teacher support and student engagement in higher education. This study aims to enhance the understanding among researchers in psychology and education regarding the correlation between perceived teacher support and student engagement within higher education settings. Additionally, it explores theoretical frameworks and methods (including measurement tools, data analysis, and results) that can inform future research in this domain.

The practical objective of this study is to maintain motivation among higher education students toward learning, emphasizing the critical need for higher education institutions to equip students with the skills required to meet societal demands. By improving teacher support, educators can significantly contribute to fostering student engagement. This literature review is intended to help researchers understand validation methodologies and guide future investigations into interventions, solutions, and strategies designed to enhance the quality of teacher-student relationships, thereby promoting student engagement in higher education settings.

## Methods

We conducted a systematic literature review of empirical cross-sectional studies on perceived teacher support and student engagement among higher education students, covering publications from January 2014 to May 2024. The decision to employ a systematic literature review is in line with the methodology recommended by Page et al. [27]. This review aims to provide a comprehensive understanding of the foundational elements and research landscape related to studies on perceived teacher support and student engagement. To conduct this study, we adhered to the Preferred Reporting Items for Systematic reviews and Meta-Analyses (PRISMA [28]) framework. This study examines various descriptive data, including the author, country, year of publication, measurement tools for perceived teacher support and student engagement, number of participants, and potential mediating and moderating factors affecting the relationship between perceived teacher support and student engagement in higher education. The identified factors were categorized into mediators and moderators related to students’ personal characteristics and school environment. Outcome measures that lacked information on these factors or unclear research patterns were excluded from the analysis.

### Search strategy

The literature search was conducted via databases such as ProQuest, ERIC, APA Psycnet, Scopus, PubMed, and EBSCOHost, which were accessed between 1 and 3 May 2024. The databases were selected for their reliability, journal indexing, and comprehensive coverage of psychology and education fields. We included peer-reviewed journal articles published from January 2014 to May 2024. Three key search terms used in the databases were “perceived teacher support” “student engagement” and “higher education.” Although similar terms such as “involvement” and “participation” appear in the literature, we focused exclusively on articles using the term “engagement” in the abstract, anticipating a direct and relevant connection to the concept of student engagement. For the ProQuest databases, we use an additional filter to limit them to academic journals, education and psychology.

We incorporated alternative terms and phrases for each key variable to broaden the search results. For Teacher Support, terms such as “perceived teacher support,” “teacher support,” “teacher-student relationship,” and “teacher autonomy support” were included. These terms were chosen to encompass a wide range of studies addressing different aspects of teacher support as experienced by students. Similarly, for Student Engagement, alternative terms like “student engagement,” “academic engagement,” “learning engagement,” and “student involvement” were utilized. These terms ensured the inclusion of studies focusing on diverse dimensions of student engagement in higher education. Finally, to capture studies specific to the higher education context, we used terms like “university students,” “higher education students,” and “college students” under the variable College Students. By combining these terms using Boolean operators such as AND, the search strategy was designed to comprehensively cover relevant literature while maintaining specificity to the topic.

### Inclusion and exclusion criteria

To facilitate the search for relevant articles, we employed a protocol based on the population, interventions, comparator, outcomes, and study (PICOS) framework. The inclusion and exclusion criteria were established to ensure the relevance and quality of studies included in this systematic review. Studies were included if they focused on higher education, university, or college students, specifically examining factors related to perceived teacher support and student engagement. Only research employing quantitative, cross-sectional methods was considered, and the studies had to be original research articles published in English between 2014 and 2024. Furthermore, only studies published in peer-reviewed and accredited journals were included to ensure academic rigor.

On the other hand, studies were excluded if they did not present a complete research report, including the introduction, theoretical framework, methodology, results, and discussion. Duplicate studies retrieved from different databases were also excluded. Additionally, studies utilizing methods other than quantitative cross-sectional approaches, such as qualitative studies, experiments, scoping reviews, systematic reviews, or meta-analyses, were not considered. Research that did not focus on higher education students or failed to specifically address the Student Engagement model or the Autonomy, Structure, and Involvement dimensions of Perceived Teacher Support was also excluded. Lastly, papers that emphasized other forms of support, such as parental support, family support, teaching style, work engagement, or teacher engagement, were omitted, as these topics were outside the scope of the review.

### Screening process

We conducted an extensive search of six databases—ProQuest, ERIC APA Psycnet, Scopus, PubMed, and EBSCOHost—accessed between 1 and 3 May 2024. The total number of articles we collected was 1095. Initially, we collected a total of 1,095 articles. Using Mendeley’s automatic function, we identified and removed 31 duplicate articles. The remaining references were then converted into an Excel file. In the first stage, the reviewers independently screened the titles and abstracts on the basis of the inclusion and exclusion criteria, narrowing the selection to 385 articles. In the second stage, reviewers conducted a full-text screening. Full-text articles that met the inclusion criteria were extracted into an Excel table by the primary author (KP) and validated by the second, third, and fourth authors (SC, FYL, ZRH).

## Results

**Fig. 1:**
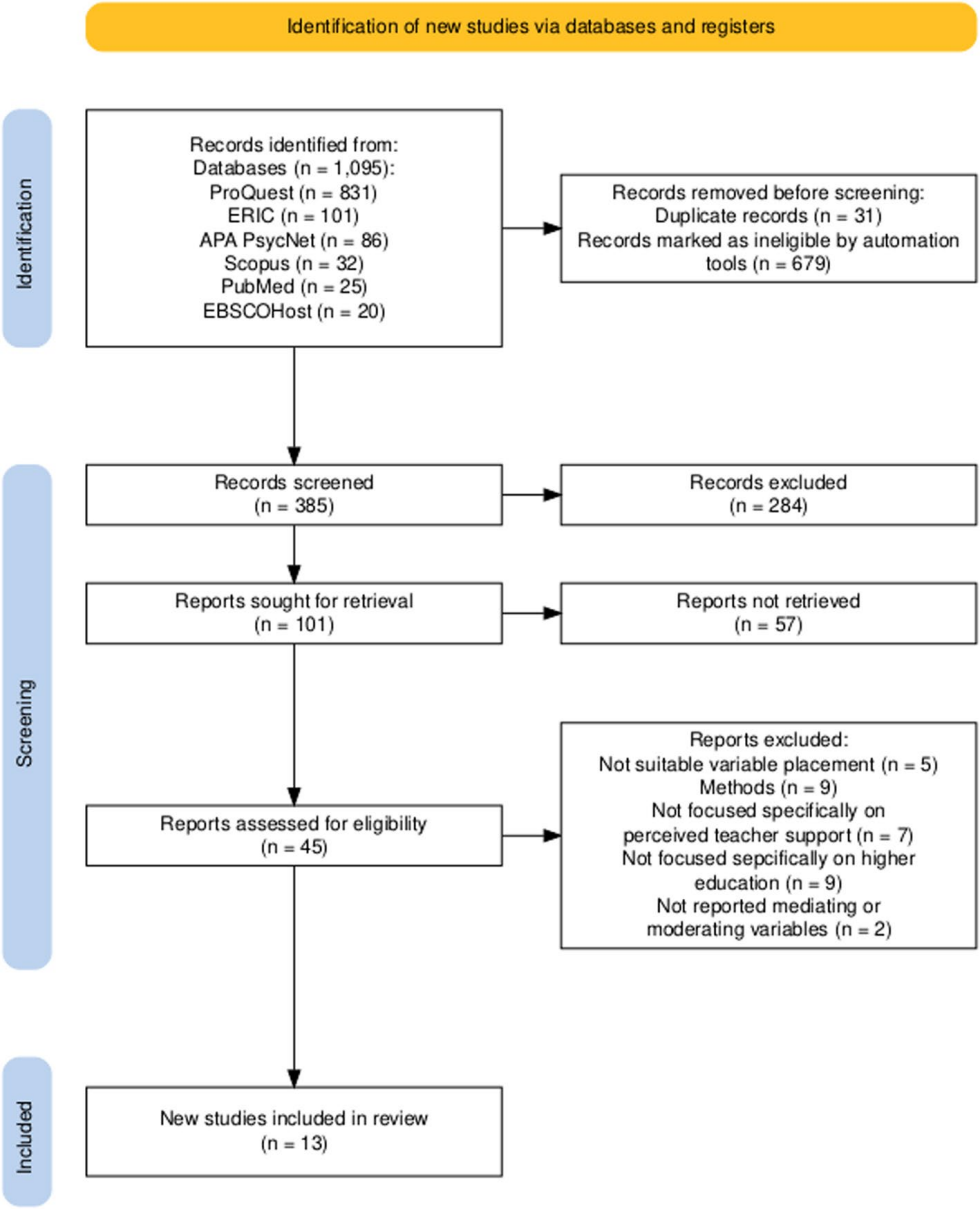
PRISMA flow diagram. Notes: Adapted from Page MJ, McKenzie JE, Bossuyt PM, etc. The PRISMA 2020 statement: An updated guideline for reporting systematic reviews. The BMJ. 2021;372

 Ultimately, 13 articles were included in the final synthesis. The detailed process is depicted in the PRISMA flow diagram shown in Fig. 1. Number of countries and years for researchers interested in the relationship between perceived teacher support and student engagement in higher education contexts.

### Creative commons

Among the 13 articles that met the inclusion criteria, several countries were represented in the studies. The most frequently studied country was China, with 11 articles (84.6%), followed by Malaysia, and Vietnam, each contributing 1 article (7.7%).

### Summary of 13 studies

Based on the data presented in Table 1, several variables have been identified as potential mediators and moderators in the relationship between perceived teacher support and student engagement. The variables under study are categorized into two primary domains: students’ personal characteristics and school environment. Students’ personal characteristics mediating variables, including behavioral engagement, personal best goals, expectancy belief, positive emotions, and basic psychological needs satisfaction, exhibit full mediation effects. In contrast, other students’ personal characteristics mediators, such as learning motivation, self-efficacy, professional commitment, enjoyment, self-regulated learning, and learning drive, demonstrate partial mediation effects. Notably, school environments mediating factors, such as teachers’ harmony, peer support, helping behavior, and the learning climate solely exhibit full mediation effects, with no instances of partial mediation effects observed.

**Table 1:** Mediating and moderating variables between perceived teacher support and student engagement

No	Authors (Year), Country	Mediating / Moderating Variables	Categorization
1	Luan et al. (2020) [28], China	Mediating: Behavioral engagement	Students’ personal characteristics mediator (full mediation)
2	Abderrahim et al.(2020) [29], Malaysia	Mediating: Personal Best Goals	Students’ personal characteristics mediator (full mediation)
3	Chen et al. (2022) [30], China	Mediating: Learning Motivation Moderating: Class Climate	Students’ personal characteristics mediator (partial mediation) School environment mediator (strengthening the relationship)
4	Feng et al. (2023) [31]China	Mediating: Students’ ICT Self-Efficacy	Students’ personal characteristics mediator (partial mediation)
5	Zhou et al. (2023) [32], China	Mediating: Academic self-efficacy & professional commitment.	Students’ personal characteristics mediator (partial mediation)
6	Haoting Li (2023) [33], China	Mediating: Foreign language enjoyment	Students’ personal characteristics mediator (partial mediation)
7	Miao et al. (2023) [34], China	Mediating: Self-efficacy and Self-regulated learning	Students’ personal characteristics mediator (partial mediation)
8	Xu et al. (2023) [26], China	Mediating: Basic psychological needs satisfaction & learning drive. Moderating: Optimistic attribution style	Learning drive: students’ personal characteristics mediator (partial mediation) Basic psychological needs satisfaction: students’ personal characteristics mediator (chain mediation) Students’ personal characteristics moderator (strengthening the relationship)
9	Liu Yang (2024) [35], China	Mediating: teachers’ harmony & students’ peer support	School environment mediator (full mediation)
10	Chen et al. (2024) [36], China	Mediating: Helping behavior & Green building learning climate	School environment mediator (full mediation)
11	Vo et al. (2024) [37], Vietnam	Mediating: expectancy belief - value belief	Expectancy belief: students’ personal characteristics mediator (full mediation) Task value belief (Students’ personal characteristics mediator) : no mediation effect
12	Jia et al. (2024) [38], China	Mediating: students emotions	Students’ personal characteristics mediator (positive emotions: full mediation, negative emotions: no mediation effect)
13	Zhou et al. (2022) [39], China.	Mediating: basic psychological needs satisfaction.	Students’ personal characteristics mediator (full mediation)

Some researchers have opted to investigate student engagement, a multidimensional concept, separately to identify which dimensions of engagement are most strongly correlated. Additionally, some researchers have designated certain dimensions as mediators while positioning others as dependent variables. Consequently, it is assumed that perceived teacher support does not uniformly impact student engagement as a whole; instead, it may influence different stages and correlations. For example, teacher support might initially be related to behavioral engagement, which subsequently affects other dimensions of engagement [29].

The development of student engagement variables has been significantly influenced by the theory proposed by Fredricks et al. [30], which conceptualizes student engagement as a multidimensional construct encompassing three dimensions: behavioral engagement, emotional engagement, and cognitive engagement. This framework remains the most widely utilized framework by researchers, accounting for 46.2%% (*n* = 6) of the studies. As the theory of student engagement evolved, some researchers introduced a fourth dimension, agentic engagement, expanding the construct to include behavioral, emotional, cognitive, and agentic engagement [31, 32, 40]. This expanded model is represented in 15.4% (*n* = 2) of the studies. Another four dimensions of engagement are behavioral, emotional, cognitive, and social engagement from Fredricks et al., 2016 are 15.4% (*n* = 2). The concept of engagement in education is also influenced by the framework of work engagement proposed by Schaufeli et al. [33] (*n* = 1; 7.7%). Two other studies (15.4%) employed different theoretical foundations, such as motivation and participatory [41].

The conceptualizations of perceived teacher support exhibit considerable variation. To address the broad variability in the concept of perceived teacher support, we categorized it into three theoretical frameworks in relation to student engagement: TS pattern, TNS pattern, and social cognitive theory. “TS pattern” (*n* = 3; 23.1%) [26, 34, 35, 42] describes the direct relationship where perceived teacher support influences student engagement (perceived teacher support ◊ student engagement). This pattern posits that teacher support is directly associated with internal factors, such as motivation, learning behavior, and engagement. In contrast, the second perspective draws on Self-Determination Theory (SDT) [11] called “TNS pattern” which highlights a pathway in perceived teacher support enhances student engagement by fulfilling basic psychological needs (perceived teacher support → basic psychological needs satisfaction → student engagement). Thereby emphasizing the importance of addressing these needs in the educational context. This pattern suggesting that teacher support fulfills students’ basic psychological needs, subsequently influencing other internal attributes as a TNS pattern (*n* = 8; 61.5%). Lastly, a smaller subset of views (*n* = 2; 15.4%) is grounded in Social Cognitive Theory, which emphasizes the role of the social environment in shaping human behavior.

For example, teacher social support functions as both an instructional behavior and an interpersonal tool to help students feel valued, engaged, and motivated (TS pattern). Consequently, when students perceive teacher support positively, they tend to have more favorable emotions toward their academic activities. Roorda et al. [36] and Ruzek et al. [37] further argued that educators who offer emotional support exhibit traits such as attentiveness, empathy, and kindness toward their students, along with a genuine interest in their attitudes, emotions, and actions. In essence, emotional support from teachers is characterized by a nurturing and positive relationship between educators and learners, which can significantly enhance students’ motivation, engagement, and sense of inclusion. Similarly, Marchant et al. [38] reported that teachers’ positive responsiveness to students’ needs enhances their overall motivation, perceived self-efficacy, and academic performance. This positive teacher‒student relationship helps fulfill students’ needs, as further explained by SDT (TNS pattern). SDT posits that satisfying three basic human needs—autonomy, competence, and relatedness—can foster students’ psychological growth, including motivation and engagement [39, 43]. Therefore, teacher support can address students’ basic psychological needs through these three interrelated but independent dimensions [39]. Despite the differing views on the concept of teacher support, these studies consistently indicate that teacher support correlates with student engagement, both directly and indirectly.

Information about the measurement tools used in examining the variables of Perceived Teacher Support and Student Engagement are presented in the Table 2 below. We present only some of the most popular instruments that have been used in previous studies.

**Table 2:** Frequently used instruments

Dimensions of measurement	Name of instruments (authors) and dimensions of student engagement	Study examples
Perceived teacher support	Learning Climate Questionnaire (LCQ, Williams and Deci, 1996), *n* = 3	Personal Best Goals: Do they Mediate the Relationship between Teacher Autonomy Support and Student Engagement? [29]
Engagement	Online English Learning Engagement (OELE) Survey adapted from Math and Science Engagement Scale (Wang, 2016), *n* = 2. Student engagement dimensions (Fredricks et al., 2016): - Behavioral engagement - Emotional engagement - Cognitive engagement - Social engagement	Exploring the role of online EFL learners’ perceived social support in their learning engagement: a structural equation model [28].
	Students’ Engagement VS Disaffection, (Skinner et al., 2008), *n* = 2. Student engagement dimensions (Skinner et al., 2008) - Behavioral engagement - Emotional engagement - Cognitive engagement	The relationship between perceived teacher support and student engagement among higher vocational students: A moderated mediation model [26]. Personal Best Goals: Do they Mediate the Relationship between Teacher Autonomy Support and Student Engagement? [29]
	Students engagement during learning activities scale (Reeve & Tseng, 2011), *n* = 2 Student engagement dimensions: - Behavioral engagement - Emotional engagement - Cognitive engagement - Agentic engagement	Chinese College Students’ Perceived Teacher Autonomy Support and Engagement: A Moderated Mediation Model [30].
	Utrecht Work Engagement Scale (UWES), (Schaufeli, 2017), *n* = 2 Student engagement dimensions (Schaufeli, 2017): - Vigor - Dedication - Absorption	Enhancing emotional health and engagement in Chinese English language learners: an approach from teachers’ autonomy- supportive behavior, teachers’ harmony, and peer support in a two-sample study [35].

### Theories, data analysis, and results of 13 studies

As shown in Table 3 below, the results indicate that the number of participants in these studies varies, with a total of 8425 students across all research, ranging from a minimum of 266 to a maximum of 1517 university students. The participants were all students currently attending university, college, or EFL (English as a Foreign Language) education. All the articles discussed employed a cross-sectional study design with self-report questionnaires. After data collection, researchers screened for valid and invalid questionnaires, retaining only the valid questionnaires for analysis. Initially, descriptive statistical analysis was performed to summarize the basic characteristics of the data, while Pearson correlation analysis was conducted to examine the relationships between variables. For validity testing, Confirmatory Factor Analysis (CFA) was used in 69.2% (*n* = 9) of the studies, aligning with its role in verifying preexisting factor structures within the dataset. Moreover, Exploratory Factor Analysis (EFA) was employed in 6.3% (*n* = 1) of the studies, which is suitable for exploring underlying factor structures in the absence or prior hypotheses. The remaining articles (23.1%; *n* = 3) did not report validity analyses. The structural equation modeling (SEM) approach was the most frequently used model-testing technique, appearing in 53.8% (*n* = 7) of the studies. SEM is widely valued for its capacity to assess complex relationships among multiple variables simultaneously, making it particularly useful in validating theoretical models. The remaining 30.8% (*n* = 4) used path analysis PROCESS on SPSS. Robust Maximum Likelihood (RML) was utilized in 7.7% (*n* = 1) of the studies for parameter estimation under non-normality conditions, and the Multiple Indicator Multiple Causes (MIMIC), was also applied in 7.7% (*n* = 1) of the studies, serving as a model selection criterion. The specific application of the MMIC, however, may benefit from further clarification because of its less common use in standard psychometric research (Table 3).

**Table 3:** Theories, measurement scale, analysis methods, results

SOURCES	Theories	Participants	Tools	Data Analysis	Results
1.Exploring the role of online EFL learners’ perceived social support in their learning engagement: a structural equation model [28].	Teachers support (Liu et al., 2018) [44] : - Academic support - Emotional support (Ecological systems theory) **“TS Pattern”** Student engagement (Fredricks et al., 2016) [45]: - Behavioral engagement - Emotional engagement - Cognitive engagement - Social engagement	615 first-year undergraduate Students. 432 males (70.2%) and 183 females (29.8%).	Perceived Social Support-English Learning (PSS-EL) survey. Adapted from Students’ perceptions of being offered academic guidance and resources (Federici & Skaalvik, 2014). Online English Learning Engagement (OELE) survey. Adapted from the measurement developed by Wang et al. (2016) (Math and Science Engagement)	CFA (validity and reliability) SEM (measurement of the structure relationships among all the constructs). Path Analysis	Behavioral engagement completely mediated the relationship among perceived teacher support and cognitive, emotional, social engagement.
2. Personal best goals: Do they mediate the relationship between teacher autonomy support and student engagement? [29]	Self-Determination Theory (SDT): Teacher Autonomy Support (TAS) is defined as the degree to which the teachers take the target students’ perspective and act in ways that enhance self- choice, self-initiation, volitional behaviours, sense of psychological freedom, and self-endorsement of the undertaking behaviors (Deci & Ryan, 2000 [11]) “**TNS Pattern**” Student engagement (Fredricks, Blumenfeld, & Paris, 2004 [46]): - Behavioral - Cognitive - Emotional	266 undergraduates from a government university in northern Malaysia. 223 females (83.8%) and 43 males (16.2%)	The Learning Climate Questionnaire (LCQ) Questionnaire (Williams & Deci, 1996) [47] Student Engagement versus Disaffection (Skinner et al., 2009) [48]	CFA SEM	Personal best goals has a full mediating effects between perceived teacher autonomy support and student engagement.
3. Chinese College Students’ Perceived Teacher Autonomy Support and Engagement: A Moderated Mediation Model [30]	Perceived Autonomy Support: Providing choices to students, fostering understanding and interest in students, allowing criticism and independent thinking from students (Assor et al., 2002 [49]) “**TNS Pattern**” Student Engagement: - Behavior - Cognitive - Emotional - Agentic (Reeve & Tseng, 2011 [50]; Wang et al., 2016 [51])	1517 valid questionnaires from Chinese college students: - 342 (22.5%) male - 1175 (77.5%) female	Student engagement: Students’ Engagement During Learning Activities Scale, Reeve and Tseng (2011 [52]) Autonomy support: Autonomy Enhancement Scales, Assor et al. (2002) [49]	CFA Path analysis using PROCESS SPSS	Perceived autonomy support (PAS) positively and significantly affected student engagement (SE). Learning motivation (LM) partially mediates association between PAS & SE among college students. Class Climate (CC) positively moderates the effect of PAS on LM.
4. The Association between Perceived Teacher Support, Students’ ICT Self-Efficacy, and Online English Academic Engagement in the Blended Learning Context [31].	Teacher Support: - emotional, - instrumental, - appraisal, - informational support (Ryan & Patrick, 2001 [53]) “**TS pattern**” Academic Engagement: - emotion engagement - behavior engagement - cognitive engagement - social engagement	960 students	Perceived Teacher Support Scale (Patrick et al., 2007) [54] Student Engagement: Math and Science Engagement Scales (Wang et al., 2016) [55] Online English academic engagement scale (Luan et al., 2022) [56]	Reliability: Cronbach’s alpha Mediating role: multiple linear regression analysis	There is a correlation between perceived teacher support and student involvement in foreign language learning in an E-learning environment. ICT self-efficacy is a strong mediator between perceived teacher support and student involvement.
5. A study on the relationship between higher vocational students’ perceived teacher support and learning engagement: The chain mediation of academic self-efficacy and professional commitment [32]	Perceived teacher support: learning climate **“TS pattern”** Learning engagement: - class discussion, - participation in group assignments, - communication with teachers and peers, - self-directed learning and involvement in school clubs and organizations (Unclear dimensions)	387 higher vocational students: Male : 119 (31%) Female : 268 (69%)	Perceived Teacher Support: Learning Climate Questionnaire (LCQ) [47] Learning Engagement: Learning Engagement Scale of Students in Higher Vocational Colleges	AVE (reliability and validity) Path Analysis	Teacher support can not only directly affect students’ learning engagement but also indirectly affect students’ academic self-efficacy and career commitment
6. Perceived teacher‒student relationship and growth mindset as predictors of student engagement in foreign student engagement in foreign language learning: the mediating role of foreign language enjoyment [33]	Teacher-student relationship: self- determination theory (Deci and Ryan, 2000 [57]), - autonomy - competence - relatedness “**TNS Pattern**” Student engagement: - behavioral - emotional - cognitive - agentic (Derakhshan et al., 2022 [58]).	413 Chinese EFL Learner: Male 188 (45.5%) Female 225 (54.5%)	Perceived teacher – student relationship scale (Wang and Wang, 2002) Student engagement scale (Reeve, 2013)	CFA SEM	Perceived teacher student relationship and foreign language enjoyment have a direct effect on student engagement. Growth mindset has an indirect effect on student engagement through the mediation of foreign language enjoyment.
7. Teacher Autonomy Support Influence on Online Learning Engagement: The Mediating Roles of Self-Efficacy and Self-Regulated Learning [34].	Learning engagement: - behavioral - emotional - cognitive (Fredricks et al., 2004 [46]) Teacher support: Autonomy: give a reasonable explanation, avoid those controlling words and reduce the unnecessary pressure they put on them (Lee et al., 2015 [59]) “**TNS Pattern**”	492 university students	Teacher autonomy support questionnaire (Jang et al., 2012) [60] Learning Engagement Scale (Sun and Rueda, 2012) [58]	CFA Path Analysis PROCESS SPSS	The more students have positive perceived teacher support, it is easier for them to increase self-efficacy, start entering the self-regulated learning stage, and increase learning engagement.
8. The relationship between perceived teacher support and student engagement among higher vocational students: A moderated mediation model [26].	Perceived teacher support: motivation for mastery, learning enjoyment, and academic success, and exhibit behaviors in task (Fraser and Fisher, 1982 [61]). Basic psychological needs satisfaction: Self-determination theory (SDT): - autonomy - competence - relatedness (Deci and Ryan, 2002 [62]) “**TNS Pattern**” Student engagement: - behavioral - emotional - cognitive (Fredricks et al., 2004 [46])	1136 students: Male: 626 (69.7%) Female: 405 (39.3%)	Perceived teacher support: Revised teacher support scale designed by Chi (Chinese version, 2017) [63] Student engagement: The student engagement scale (Skinner et al., 2008) [48]	Mediation effect : SEM Moderation effect: Path Analysis PROCESS, SPSS	Perceived teacher support has significant positive effects on basic psychological needs satisfaction, learning drive and student engagement; Basic psychological needs satisfaction has a significant positive impact on learning drive; learning drive has a significant positive impact on student engagement; However, the impact of basic psychological needs satisfaction on student engagement is not significant. Learning drive partially mediates the relationship between perceived teacher support and student engagement. Basic psychological needs satisfaction and learning drive have a multiple chain mediating effect between perceived teacher support and student engagement. OAS_P style significantly moderates the relationship between basic psychological needs satisfaction and learning drive, learning drive and student engagement.
9. Enhancing emotional health and engagement in Chinese English language learners: an approach from teachers’ autonomy- supportive behavior, teachers’ harmony, and peer support in a two-sample study [35]	Teachers’ autonomy supportive behavior: - learning support, - emotional support, - ability support (Hu et al., 2023 [64]) “**TNS Pattern**” Academic engagement: - behavioral - emotional - cognitive (Fredricks et al., 2004)	Students: 389 Teachers: 68	Teachers’ autonomy supportive behavior: Learning Climate Questionnaire (Black and Deci, 2000) [47]. Student engagement: Utrecht Work Engagement Scale (UWES–3) (Schaufeli et al., 2017). [65]	Mediation analysis: MIMIC procedure, PROCESS	Teachers’ autonomy supportive behavior is the strong influence for student engagement. Peer support and teachers’ harmony also significant predictors for student engagement.
10. The Multilevel Chain Mediating Mechanism of College Faculty’s Felt Responsibility on Students’ Engagement in Green Building Learning [36]	Learning engagement: work engagement concept by Schaufeli et al.: - dedication - concentration - vitality Teachers’ felt responsibility: - perception of professional responsibility - emotional engagement - awareness of professional responsibility - professional responsible behavior. Lauermann et al., 2011 [66] “**Social cognitive Theory**”	832 students	Perception of responsibility among university teachers (Zhou Xihua, Morison et al., 1999) [67] Engagement in green building learning: UWES-S [65] (translated by Fang Laitan et al., 2008)	Validation factor analysis (MCFA) Model testing: robust maximum likelihood (MLR) using Mplus 8.3 software	Helping behavior can mediate students engagement Learning climate can indirectly influence students engagement through helping behavior Direct effect of teachers’ perceived responsibility on students engagement is not significant.
11. Online learning environment and student engagement: the mediating role of expectancy and task value beliefs [37].	Student engagement: - affective - behavioral - cognitive (Fredricks et al. [46]., 2004; Wang et al., 2020 [68]) Teacher support (social support): - autonomy - competence - relatedness (Wang et al., 2020) [68] “**TNS Pattern**”	351 students Male : 113 (32.5%) Female: 237 (67.5%)	Teacher support scale: approachable and emotionally supportive (Kaufmann et al., 2016) [69] Student engagement scale: 12 item scale from Hoi and Hang (2021a, 2021b) [70]	CFA SEM	Ecological perspective (i.e.: Teacher support) influence student engagement via expectancy belief, rather than task value belief.
12. Effect of teacher social support on students’ emotions and learning engagement: a U.S.-Chinese classroom investigation [38].	Teachers’ social support: - emotional, - informational - self-esteem, - network Xu and Burleson (2001) [71] Student engagement: - motivation - participatory (Mazer, 2012 [72])	362 students US: 164 China: 198	Teacher social support: revised Social Support Scale (Xu & Burleson, 2001 [71]). Students’ engagement: Student Engagement Scale (Mazer, 2012 [72])	Descriptive statistics & Pearson correlation: measure the correlations between the variables Independent t-test: comparing US and Chinese students responses. Path Analysis PROCESS SPSS	Teacher support (received informational, emotional, and esteem support) are the same among US and China culture. US teachers provide significantly higher levels of network support rather than Chinese teachers. U.S. students reported a slightly higher level of positive emotions, particularly being determined and attentive in class Chinese students experienced higher negative emotions in class than U.S. students, which involved being upset, guilty, nervous, and afraid Higher perceived teacher social support, can help students feel more positive(emotion) about the class. Positive emotions, mediate the relationship between perceived social teacher support and students engagement.
13. Effects of perceived teacher support on motivation and engagement amongst Chinese college students: Need satisfaction as the mediator [39].	Student engagement: - behavioral - emotional - cognitive (Fredricks et al., 2004 [46]) Perceived Teacher Support: - autonomy - structure - involvement (Reeve et al., 2004 [73]; Ryan and Deci, 2020 [57]) “**TNS Pattern**”	705 students Female: 79.7% Male: 20.3%	PTS: Teacher as Social Context scale (TASC; Belmont et al., 1992 [74]; Haerens et al., 2013 [75]) Class Engagement: Individual Self-Report Engagement scale was used (Jang et al., 2010 [25]).	CFA Model : SEM Examine hypothesis: maximum likelihood estimation with robust standard errors (MLR)	Autonomy support and involvement (perceived teacher support) positively linked basic needs satisfaction, autonomous motivation, controlled motivation, class engagement. Autonomous motivation affect engagement. Need satisfaction-autonomous motivation statistically mediated the association between involvement support and class engagement. Need satisfaction is a positive predictor of autonomous motivation, controlled motivation, and class engagement. Autonomous motivation was found to be a predictive factor of class engagement. Higher level SDT, higher level of engagement.

*CFA* Confirmatory Factor Analysis, *EFA *Exploratory Factor Analysis*, SEM *Structural Equation Modelling, *AVE* Average Variance Extracted, *SPSS* Statistical Package for the Social Sciences, *MIMIC* Multiple Indicators Multiple Causes, *MCFA* Multigroup Confirmatory Factor Analysis

## Discussion

Our review demonstrated that teacher support influences student engagement. Various factors affect the relationship between perceived teacher support and student engagement. For the mediating factors, we categorized the findings into students’ personal characteristics, and school environment. The students’ personal characteristics including the satisfaction of basic psychological needs, behavioral engagement, self-efficacy, personal best goals, learning motivation, learning drive, commitment, self-regulated learning, enjoyment, student emotions, and expectancy beliefs. The school environment variables such as teachers’ harmony, peer support among students, helping behavior, and the overall learning climate. The variables that fully mediate the relationship include teachers’ harmony, students’ peer support, basic psychological needs satisfaction, positive students’ emotions, expectancy belief, helping behavior, the learning climate, emotional intelligence, personal best goals, and behavioral engagement. Meanwhile, the variables that partially mediate the relationship are learning drive, self-efficacy, self-regulated learning, enjoyment, learning motivation, and professional commitment. For the moderating role, we also categorized the findings into students’ personal characteristics and school environment. The optimistic attribution style is a students’ personal characteristics moderating variable, and the class climate is a school environment moderating variable. Both of these moderating variables can strengthen the relationship between perceived teacher support and student engagement.

According to SDT, teachers can increase student engagement in learning by nurturing internal motivators such as self-efficacy, personal interests, values, preferences, and goals [34]. In the context of SDT, basic psychological needs such as the need for autonomy, competence, and relatedness are usually considered to play important roles in increasing student motivation and engagement. This relationship is not direct and may require other additional factors for this pathway to be significant. By adding other mediators, such as optimism, this pathway relationship becomes significant [26].

Scott and Walczak [76] assert that effective educator support can fulfill students’ essential psychological needs for autonomy, competence, and relatedness. When these needs are satisfied, internal factors such as self-confidence and self-belief are enhanced [34]. This is consistent with Deci and Ryan [11], who described self-efficacy as an emotional state reflecting one’s perception of one’s capabilities. A stronger belief in their abilities drives students to set and achieve goals, thereby improving their performance. Additionally, Zhou and Wu [77] reported that students who perceive emotional support from teachers exhibit increased learning participation, emotional satisfaction, and learning confidence, which fosters more active engagement in acquiring new knowledge.”

Self-efficacy is closely related to expectancy belief, as both can enhance confidence in successfully completing tasks, thereby promoting behavioral and emotional engagement. Research by Eccles and Wigfield [78] indicates that students who perceive their teachers as supportive (e.g., showing care, concern, respect, and understanding) employ more advanced cognitive strategies and engage more deeply in the online learning process, demonstrating greater cognitive engagement [79]. Additionally, the concept of personal best goals is crucial in explaining the motivational drivers behind activities in various contexts [80]. Personal best goals are also predictive of academic outcomes, such as student engagement [76], achievement [81], and task accomplishment [82].

Another students’ personal characteristic mediator is professional commitment; students who demonstrate greater professional commitment are often more dedicated to their academic work [83]. Zhou et al. reported that teacher support can not only directly affect students’ engagement but also indirectly affect their academic self-efficacy and career/professional commitment [39]. Students with strong professional commitment tend to exhibit greater academic dedication. Professional commitment often mediates the relationship between environmental factors and students’ learning outcomes. Additionally, a sense of psychological attachment, such as a sense of belonging, is critical for college students and plays a key role in fostering learning engagement. Given these findings, professional commitment can be regarded as an intermediary variable in understanding students’ learning engagement. Self-regulated learning, as defined by Zimmerman [84], involves a set of self-generated thoughts, emotions, and behaviors aimed at achieving a goal. Lai and Hwang [85] identified factors such as goal setting, time management, task strategies, help-seeking, and the environment as dimensions of self-regulated learning, all of which contribute to learners’ emotional and behavioral engagement and learning performance. Studies have shown that teachers’ support for autonomy significantly impacts students’ self-regulated learning. For example, Won and Yu [86] demonstrated that students’ perceptions of teacher autonomy support positively influence their support for teachers. Similarly, Grijalva-Quiñonez et al. [87] reported that autonomy is significantly and positively related to students’ self-regulation. Miao et al. reported that teacher support for autonomy serves as a strong predictor of students’ self-efficacy. Self-efficacy was subsequently shown to significantly influence students’ self-regulated learning (SRL), which, in turn, enhanced their engagement in online learning. Unlike prior studies that examined these variables in a simultaneous model, Miao et al.‘s findings reveal a sequential pathway, where self-regulated learning (SRL) potentially acts as a mediating link. This suggests that SRL may function as a transitional mechanism through which online learning engagement is indirectly facilitated [34].

Jia and Cheng reported that students reported slightly elevated levels of positive emotions, particularly in feelings of motivation and focus during class. These findings support the idea that teachers who provide social support—such as offering problem-solving guidance, enhancing students’ self-esteem, and connecting them with social resources—can positively impact students’ feelings toward class. Furthermore, experiencing positive emotions was found to increase student engagement, encouraging behaviors such as active participation in discussions, attentive listening, and reflection on how class material applies to personal and professional contexts [38]. Other research has also revealed that teachers’ verbal and non-verbal behaviors significantly influence students’ emotions, impacting whether they avoid or approach learning tasks. Experiencing positive emotions such as pleasure can enhance students’ approach behaviors, such as attending classes, whereas feeling empowered can increase their confidence, and passion can intensify their academic emotions [88, 89].

Motivation plays a crucial role as a student personal characteristic mediator. Findings [30] indicate that the school environment (scaffolding) significantly influences motivation, which subsequently affects behavioral, emotional, and cognitive engagement. When students are motivated, they are better able to manage negative emotions, as research suggests that emotional disengagement often correlates with feelings of boredom. Motivated students are more likely to employ cognitive techniques, such as elaboration and organization, to complete their assignments. They favor deeper, more complex learning strategies over surface-level strategies, which supports higher academic achievement and success. Additionally, motivated students demonstrate greater behavioral engagement by investing more time and actively participating in learning activities. According to Chen et al. [30], teachers have the unique ability to increase students’ positive emotions, thereby increasing their motivation toward school activities, which in turn positively influences their academic performance. In line with research from Skinner et al. [10], Reeve [50], and Wang and Eccles [51], the school environment plays a crucial role in shaping motivation and influencing emotional, behavioral, and cognitive engagement. SDT research frequently associates teacher support with internal motivation [783]. Numerous studies have demonstrated that teacher autonomy support profoundly impacts learning motivation [53, 90–92]. Additionally, increased motivation has been shown to enhance student engagement [11, 51, 93].

Moreover, enjoyment was found to have a direct effect on student engagement. When teachers foster a positive and enjoyable learning environment, students’ intrinsic motivation tends to increase, enhancing their engagement in learning a foreign language. In support of this, Dincer et al. [94] reported that when students feel that their needs are met by supportive instructors, they report greater autonomy, competence, connection with others, motivation, and academic success. As discussed by Dewaele and MacIntyre [95], pleasure-related motivation further supports this finding. Students receiving emotional support can foster a positive and supportive environment through successful teacher‒student interactions. Such interactions enhance their ability to manage fatigue and expand their emotional capacities, resulting in increased social, emotional, behavioral, and cognitive engagement [96, 97].

Next, there are school environment factors explained. These include teachers’ harmony, students’ peer support, helping behavior, and the learning climate. Teachers’ harmony significantly contributes to improving their psychological well-being and enhancing students’ learning outcomes [98]. A harmonious environment assists teachers in managing stress and maintaining a stable psychological state, thereby fostering supportive relationships with students [99], which subsequently increases student engagement [100]. Another critical variable is students’ peer support, which is essential in promoting emotional health and engagement within the educational context [101]. Conversely, the relatively lower influence of peer support on engagement, compared with teacher behaviors that promote autonomy and harmony, indicates a more complex role for peer interactions in supporting engagement. While peer support is valuable, it may not have as strong an impact on engagement as the teacher‒student relationship does in shaping educational outcomes [35]. Teachers’ helping behaviors can motivate them to create a positive learning environment, address student needs, and facilitate the development of cooperative and supportive behaviors among students. Teachers who view it as their responsibility to establish conditions conducive to mutually supportive learning create a positive classroom climate, encouraging students to actively engage in collaborative discussions [66].

Moderating factors include both student’s personal characteristics factor, such as an optimistic attribution style, and school environment elements, such as the classroom climate. The positive dimension of an optimistic attribution style (OAS_P) plays a significant role in moderating the link between the satisfaction of basic psychological needs and learning motivation, as well as the connection between learning motivation and student engagement [26]. OAS_P will assist individuals in maintaining motivation and experiencing positive emotions such as self-confidence and self-esteem, thereby increasing their motivation. When teachers provide support, individuals perceive their behavior as highly autonomous, which activates internal motivation and fosters autonomous behavior [102]. Additionally, the classroom climate serves as a moderating variable, influencing the relationships between variables. It impacts students’ perceptions of autonomy, competence, and motivation to learn [51, 103], which in turn affects their growth and development [99]. A positive classroom climate enhances students’ pleasure, competence, and intrinsic motivation, leading to increased satisfaction, motivation, and academic achievement [51, 104, 105].

The second aim of this article review is to identify the key pillars by examining the following theoretical frameworks and research methodology employed in investigations exploring the interplay between perceived teacher support and student engagement within higher education settings. First, our findings indicate that the majority of research on this topic has been conducted in China, with only one article each from Malaysia and Vietnam. Although we searched databases for the last decade, we only found relevant articles starting in 2020. No articles were found from 2014 to 2020, despite the development of theories on teacher support and student engagement since the early 2000s. This discrepancy may be because earlier research on the relationship between teacher support and student engagement focused predominantly on younger students (elementary, secondary, and upper secondary school students) and was often conducted in classroom settings. Research at the higher education level has only gained traction since 2020. Moreover, previous studies have suggested that student engagement is a promising intervention to increase academic achievement, particularly among secondary school students [45, 106]. However, many of these earlier studies employed qualitative and longitudinal methods, which did not meet our inclusion criteria for cross-sectional quantitative research designs. Consequently, research examining the relationship between teacher support and student engagement in higher education is lacking. This highlights a critical need for further research in this area, as university students continue to require teacher support similar to that of students at other educational levels.

Second, the articles we selected predominantly employed a cross-sectional quantitative research design with self-report questionnaires. Descriptive statistics were used to describe the demographic data of the participants. Pearson correlation analysis was used to measure the strength of the relationships between variables. To assess the validity of the measurement instruments, both confirmatory factor analysis (CFA) and exploratory factor analysis (EFA) were conducted. For model testing and determining the effects of mediators and moderators, various statistical techniques have been used, including structural equation modeling (SEM), multiple indicator multiple causes (MIMIC), and the robust maximum likelihood method.

Third, regarding the theoretical framework for the student engagement variable, most researchers utilize the model proposed by Fredricks et al. [46], which includes behavioral, emotional, and cognitive engagement. This is followed by an extension of Fredricks’ theory to incorporate agentic and social engagement. Additionally, some researchers apply the concept of work engagement in educational settings. For the theories on perceived teacher support, we observed significant variation. Therefore, we categorized these theories into three groups: teacher support, which directly correlates with internal attributes (TS pattern); teacher support, which influences basic psychological needs and subsequently correlates with internal attributes (TNS pattern); and perspectives, which are based on social cognitive theory.

Some studies indicate that teacher support can directly influence student engagement (TS pattern). The quality of teacher‒student interactions is crucial for understanding student engagement, with personalized feedback and guidance significantly enhancing it [107]. High-quality teacher‒student relationships promote desired student behaviors, improve learning performance, increase closeness with instructors, and reduce conflicts between students and teachers [108]. Additionally, caring, kindness, and harmonious relationships between teachers and students fulfill students’ emotional needs, thereby fostering student development [109, 110]. Furthermore, according to Wentzel, Russell, and Baker [111], the immediate actions of teachers that encourage students’ motivation and learning behavior are critical. However, some researchers suggest alternative views, indicating that teacher support alone may not directly impact student motivation and engagement. Instead, other internal and external factors are likely necessary to influence and shape the dynamics of student engagement.

However, many studies employ SDT to explain perceived teacher support. Consequently, many studies follow the pattern of “perceived teacher support → basic psychological needs satisfaction → students’ personal characteristics and school environment mediating variables → student engagement [11, 103] (TNS pattern). Therefore, teachers suggest understanding the basic psychological needs of students to promote more positive learning behaviors and enhance engagement. The most frequently mentioned teacher support strategies in the reviewed articles are based on the premises of Ryan and Deci [11, 62] and Reeve et al. [73].

### Theoretical implications

This systematic literature review advances the understanding of student engagement by synthesizing findings on the relationship between perceived teacher support, basic psychological needs, and engagement. This study highlights the mediating role of basic psychological needs, as conceptualized in *Self-Determination Theory (SDT)*,* through a comprehensive review*, in linking teacher support to student engagement. This reinforces the theoretical perspective that fulfilling intrinsic needs for autonomy, competence, and relatedness is vital to fostering deeper engagement in higher education settings.

By consolidating findings across diverse contexts, this study further underscores the importance of considering student personal characteristics and school environments as influential factors that shape student engagement. Recognizing this complexity encourages researchers to adopt a holistic and context-sensitive approach in future studies, ensuring that both internal and external factors are accounted for when examining student engagement dynamics. Additionally, the findings of this review reveal theoretical gaps, including the need for more longitudinal, experimental, and context-specific investigations to strengthen our understanding of how teacher support and basic psychological needs interact over time to influence engagement. This synthesis serves as a theoretical foundation for advancing research on student motivation, learning outcomes, and the mechanisms that drive engagement in diverse educational contexts.

### Practical implications

This systematic literature review highlights key factors influencing the relationship between perceived teacher support and student engagement in higher education. The findings emphasize that teacher support significantly impacts internal factors, such as fulfilling students’ basic psychological needs (e.g., autonomy, competence, and relatedness) and enhancing their self-efficacy, both of which are essential for fostering engagement in university settings.

To improve student engagement in higher education, educators should prioritize cultivating strong teacher‒student relationships and fostering a positive, supportive learning climate. This can be achieved through implementing instructional strategies that address students’ psychological needs, such as encouraging autonomy through participatory learning methods, providing constructive feedback to boost competence, and building inclusive environments that foster a sense of belonging.

Additionally, policymakers and institutional leaders should consider designing professional development programs that equip higher education instructors with the knowledge and skills to create engaging learning environments. Such training initiatives can focus on recognizing and responding to diverse student needs while enhancing educators’ ability to motivate and engage students effectively. By addressing both the internal characteristics of students and external educational environments, higher education institutions can create conditions that support deeper learning engagement, ultimately contributing to improved academic outcomes and student satisfaction.

### Limitations

This study is subject to several limitations. First, the scope of the literature reviewed is restricted by specific inclusion criteria, notably limiting sources to English-language publications. Consequently, excluding non-English research may result in the omission of potentially valuable insights. It was challenging to identify all relevant studies due to the limited availability of research about perceived teacher support and student engagement in higher education contexts. However, we made every effort to include all pertinent studies within our scope. Additionally, the search was restricted to the period from 2014 to 2024, which may have resulted in the omission of earlier studies.

Second, this review exclusively includes quantitative, cross-sectional studies that capture data at a single point in time. This methodological approach limits the ability to establish causality or evaluate longitudinal impacts over time. Third, the results of this study cannot be generalized to all student populations because our search was limited to reviewing studies relevant only to higher education students and did not include primary, secondary, or high school students. We recommend that future research explore perceived teacher support and student engagement across more diverse range of student populations and methods could provide more comprehensive perspectives.

## Conclusion

To enhance student engagement and support learning success, it is essential to focus on the level of teacher support provided. This research aims to highlight the crucial role that teacher support plays in fostering both external and internal factors that enhance student engagement. We found that internal factors such as basic psychological needs satisfaction, motivation, and self-efficacy, as well as external factors such as class climate and positive relationships with teachers and peers, act as key mediators in this process. Fredricks et al.‘s multidimensional model of student engagement—comprising behavioral, emotional, and cognitive dimensions—remains a common framework in this area of study. Moreover, SDT (Ryan & Deci) is frequently used to explain teacher support. The Learning Climate Questionnaire (LCQ) is widely used to measure teacher support, but the tools for measuring student engagement vary greatly. Although significant progress has been made in exploring the connection between teacher support and student engagement, especially in recent years, research within higher education is still limited. There is a need for further exploration into the most effective measurement tools. This study provides valuable insights for improving student engagement through enhanced teacher support, offering practical implications for higher education to promote student engagement.

## Data Availability

Since this research is a systematic review, all the analyzed data were sourced from the databases used. While the datasets utilized in this study are not publicly available, they can be provided by the corresponding author upon reasonable request.
